# The Use of Biomass for Electricity Generation: A Scoping Review of Health Effects on Humans in Residential and Occupational Settings

**DOI:** 10.3390/ijerph15020354

**Published:** 2018-02-16

**Authors:** Alice Freiberg, Julia Scharfe, Vanise C. Murta, Andreas Seidler

**Affiliations:** 1 Boysen TU Dresden Graduate School, Technische Universität Dresden, 01062 Dresden, Germany; 2 Institute and Policlinic of Occupational and Social Medicine, Medical Faculty Carl Gustav Carus, Technische Universität Dresden, 01307 Dresden, Germany; julia.scharfe@gmx.de (J.S.); vanise80@hotmail.com (V.C.M.); ArbSozPH@mailbox.tu-dresden.de (A.S.)

**Keywords:** bioenergy plants, biomass, electricity generation, health impact, occupational setting, workers, residential setting, residents, scoping review

## Abstract

The utilization of biomass for power generation has become more prevalent globally. To survey the status of evidence concerning resulting health impacts and to depict potential research needs, a scoping-review was conducted. Biomass life cycle phases of interest were the conversion and combustion phases. Studies from occupational and residential settings were considered. The scoping review was conducted systematically, comprising an extensive literature search, a guided screening process, in-duplicate data extraction, and critical appraisal. Two reviewers executed most review steps. Nine articles of relevance were identified. In occupational settings of biomass plants, exposure to endotoxins and fungi might be associated with respiratory disorders. An accidental leakage of hydrogen sulfide in biogas plants may lead to fatalities or severe health impacts. Living near biomass power plants (and the accompanied odorous air pollution) may result in an increased risk for several symptoms and odor annoyance, mediated by perception about air pollution or an evaluation of a resulting health risk. The methodological quality of included studies varied a lot. Overall, the body of evidence on the topic is sparse and future high-quality research is strongly recommended.

## 1. Introduction

Climate change is predicted to have an impact on future human health globally. According to the World Health Organization, in 2030 many deaths are projected to be attributable to the consequences of climate change globally, more precisely due to childhood malnutrition (*n* = 95,000), malaria (*n* = 60,000), diarrhea (*n* = 48,000), and heat exposure for elderly people (*n* = 38,000) [1]. A measure to mitigate these effects is to reduce climate pollutants (e.g., carbon dioxide) [1]. One way to foster global decarbonization, and thus to achieve the goals of the Paris Agreement on Climate Change in 2015, is to expand renewable energy technologies [2].

Bioenergy is, amongst others, one form of renewable energy. Bioenergy is renewable, in contrast to fossil fuels, but as it relies on combustion (as opposed to most other renewable power systems), it still produces notable amounts of air pollution [3]. In 2016, the total global capacity of renewables was 2,006,202 megawatt, with a share of 109,731 megawatt produced by bioenergy [4]. The global proportion of installed capacity of bioenergy among all renewable energy technologies was 5.3%, and of electricity generation 8.6%, in 2015 [5]. The International Renewable Energy Agency remarks that there is little information available on employment data in the biogas and biomass industries [6]. Globally, 822,000 direct and 382,000 indirect jobs in solid biomass technology and biogas technology industries were counted in 2016 [6]. Note that these statistics not only comprise jobs in power generation plants, but also jobs in cooking and heating with biomass fuels.

Biomass can be utilized energetically by generating electricity, heat, and fuel. There are different research areas around human health impacts of the use of biomass for energetic purposes. Some papers examine the occurrence of respiratory diseases caused by the domestic burning of biomass for cooking and heating, primarily in developing countries [7,8,9]. Others studied health effects of pre-harvest sugar cane burning in Brazil [10,11]. These two research topics were not within the scope of this scoping review. The issue of health effects for humans caused by biomass-use for power generation (and thus by bioenergy plants) remains rather unexplored.

Different biomass fuels are utilized for bioenergy power generation, such as agricultural residues, wood chips, wood residues, specifically grown energy crops (e.g., miscanthus, switchgrass), as well as waste materials [12]. There are several types of bioenergy plants: biogas power plants, vegetable oil power plants, wood-fired power plants, wood gasification plants, power plants of the pulp industry, sewage gas power plants, and landfill gas power plants [13]. A narrative review published in 2015 investigated occupational exposures and health risks associated with biomass-based power generation [12]. Health effects at the population level remained unconsidered. This narrative review focused on direct-fired, stand-alone technologies for power generation. Only one epidemiological study that specifically dealt with the health status of workers in a straw and wood power plant was identified. Thus, the authors of the 2015 review concluded that observational studies on the topics are lacking. A scientific overview issuing health impacts of biomass-use for power generation on humans in residential settings is not available.

As a result, this scoping review was executed to take both humans in occupational settings as well as humans in residential settings into account. Further, the review process was intended to be transparent and systematic. The review focused on bioenergy plants covering the conversion phase of biomass (as preparation for power generation) as well as on the direct power generation phase (through combustion of biomass). The aims of this scoping review were to survey and illustrate the body of evidence around health effects resulting from electricity generation through biomass use on humans in residential as well as occupational settings, to identify potential research gaps, and thus to highlight the need for future research.

## 2. Materials and Methods

To achieve the aim of this research, a scoping review was conducted. The study protocol of the scoping review can be found in the International Prospective Register of Systematic Reviews (PROSPERO, registry number: CRD42016035841) [14]. Reporting of the article was supported by using the PRISMA-checklist [15], as a specific reporting guideline for scoping reviews is not yet available [16].

### 2.1. Identifying the Research Question

Based on the aims of the scoping review, two research questions were asked, a content-related one and a methodological one. The content-related one is as follows: “What health effects result from the use of biomass for electricity generation for humans in residential settings and in occupational settings”? The methodological research question reads as follows: “What is the body of evidence around health effects of the use of biomass for electricity generation on human health, and do research gaps arise”?

### 2.2. Identifying Relevant Studies

A comprehensive literature search took place to identify all relevant studies on the topic, comprising several search sources.

#### 2.2.1. Electronic Database Search

The following medical databases were searched from 2000 until 2 February 2016, and updated on 25 January 2018:

1. MEDLINE (via Ovid)
2. EMBASE (via Ovid)
3. CINAHL (via EBSCOhost)

The search strategy was designed sensitively, consisting only of terms describing the exposure and the outcome. All search strings are published in the PROSPERO study protocol [14].

#### 2.2.2. Fast-Forward Search

A fast-forward search of all finally included studies was executed with the “Cited Reference Search”-function in the Web of Science Core Collection.

#### 2.2.3. Google Scholar-Search

Google Scholar was searched with two different search strings. As this database allows for 256 spaces maximum, only the most important terms for exposure and outcome were considered. No restriction for publication date was set. Search hits were sorted by relevance, not by date. Google Scholar displays only the first 1000 hits.

#### 2.2.4. Internet-Based, Institutional Search

The following national and international (websites of) institutions were screened for relevant literature, taking the search period from 2000 onwards into account:

1. World Health Organization
2. International Labour Organization
3. European Agency for Safety and Health at Work
4. The National Institute for Occupational Safety and Health
5. Health and Safety Executive
6. Safe Work Australia
7. German Environment Agency
8. (German) Federal Ministry of Labour and Social Affairs
9. (German) Federal Ministry for the Environment, Nature Conservation, Building and Nuclear Safety
10. German Social Accident Insurance
11. (German) Federal Institute for Occupational Safety and Health
12. Deutsche Gesellschaft für Arbeits- und Sozialmedizin e. V. (no English translation available)
13. German Social Accident Insurance

#### 2.2.5. Hand Searches

Reference lists of all included studies and of topic-related key articles were screened. In addition, references found in publications which were not identified by the aforementioned searches were checked for relevance (snowball technique).

### 2.3. Study Selection

Inclusion and exclusion criteria concerning the population, exposure, outcome, and study design were defined to specify the research questions and to guide the study selection process (Table 1). Regarding the population, no age and regional restrictions were applied. Health effects of interest could have been evaluated subjectively and objectively, meaning that also subjective experiences like annoyance were considered. The decision to consider analysis as an appropriate study design was made a posteriori during the conduction if the study (after drafting the study protocol) as it was recognized that some of these contained important information relevant to the study topic. Only articles written in English and German were included. Both peer reviewed and non-peer reviewed publications were of relevance. Papers published in 2000 and later were eligible. In 2000, the German government passed the first Renewable Energies Act, which, among other incentives, increased state financial subsidies for clean energy production, to facilitate the expansion of renewables [17]. Ever since, the share of renewable energies of the primary energy consumption in Germany has increased continuously (2000: 2.9%; 2016: 12.6%) [18]. According to a press release of the German Renewable Energies Agency from 11 March 2014, more than 90 states and provinces worldwide have introduced similar funding models [19].

Titles/abstracts and full texts identified with the initial electronic database search (conducted on 2 February 2016) were screened independently by two reviewers (AF, JS). In case of disagreement, the two reviewers discussed the issue. If there remained a non-consensus, a third reviewer was consulted (AS). A decision guideline supported the reviewers during both screening phases. During title- and abstract screening, only titles and abstracts of a reference were visible. Reviewers were blinded to author names and publication year. For full text screening, excluded studies were documented, noting the reason for exclusion. Both screening phases were piloted beforehand. The proportion of agreement among the two reviewers and Cohen’s Kappa were calculated [20].

All other searches were carried out by one reviewer. If a full text seemed to be of relevance, it was screened subsequently by a second reviewer. In case of disagreement, the same procedure as described above was applied.

### 2.4. Exctracting and Presenting the Data

#### 2.4.1. Data Extraction

Data were extracted by one reviewer and checked in duplicate by a second reviewer. Data were collected in a standardized data extraction sheet, documenting information about the reference (e.g., author names, title, publication year), methods (e.g., study design, objectives, statistical methods), population (e.g., type, sample size, response, characteristics), exposure (e.g., type, measurement instrument), outcome (e.g., type, measurement instrument), results, and additional notes (e.g., conclusion of the study authors, information on funding and conflict of interest). The data extraction phase was piloted.

#### 2.4.2. Assessment of Study Methods

Initially, for each study, methodological strengths and weaknesses (taking the specific study design into account) were recorded in the data extraction sheet by one reviewer and checked by the second reviewer. In a second step, based on this evaluation, the methodological quality for different study design-specific categories was determined by one reviewer (AF). Within this scoping review, observational studies and content analyses were included. Categories for both study designs were based on checklists of the Critical Appraisals Skills Programme [21] and included the following: Reporting quality, sampling, response, eligibility of the comparison group, selection bias, information bias resulting from exposure or outcome, consideration of confounders, conflict of interest, funding, ethics committee approval, and generalization*. “Reporting quality”* was deemed of high methodological quality if sufficient information was outlined regarding study objectives, research question, setting, population, exposure, outcome parameters, research methods in general, statistical methods, and main findings. In case of random selection of participants or census recruitment, a masking of the study purpose, and the recruitment of all participants from the same population over the same period of time, *“sampling”* was of high methodological quality. The *“response”* was determined to be of high methodological quality if it was higher than 50%, or if it has been assured that a lower response had no influence on study results (e.g., proven by a non-responder analysis). For case series, judgment of the “response” was not applicable, as this study design does not intend to recruit a representative sample. For content analyses, “sampling” and “response” were not assessed since this study type investigates documents, not “real” participants. If a comparison group was available and it was eligible in relation to the study purpose, the category *“eligibility of comparison group”* was of high methodological quality. No *“selection bias”* was assigned if the categories “sampling” and “response” were of high methodological quality, thus resulting in a high methodological quality-assessment. For case series, the judgment of this category was based only on the category “sampling”, and for content analyses on the document retrieval process. No *“information bias for exposure”* was present if the risk factor was measured with valid and reliable as well as more objective than subjective instruments (high methodological quality). No “information bias for outcome” existed if the outcome parameters were also measured with valid and reliable as well as more objective than subjective instruments (high methodological quality). Moreover, subjective complaints should have been evaluated with validated questionnaires, and diseases with registers, insurance data, or doctoral diagnoses. The category *“consideration of confounders”* was of high methodological quality if regression analyses were adjusted for confounders. The category *“conflict of interest”* among the study authors was of high methodological quality if an appropriate statement was included in the study, and if no conflict of interest was obvious. A high methodological quality for *“funding”* was assigned if a study stated that it was not funded, or if funding did not lead to a conflict of interest. The category *“ethics committee approval”* was of high methodological quality if the study stated that an ethics committee approved study conduction. This category was not assessed for content analyzes, since these investigate documents, not humans. *“Generalization”* was of high methodological quality if study results could be transferred to a general population when taking study population, setting, and region into account. Overall, if the “high methodological quality”-criteria were not fulfilled, a low methodological quality was determined. An unclear methodological quality occurred if no or insufficient information for judgment of the category was given. Reasons for every judgment were documented.

#### 2.4.3. Data Analysis

After data extraction, all studies were analyzed descriptively and tabularly regarding study characteristics, study findings, and methodological aspects. For each study, results in relation to the content as well as to the method´s assessment are presented in parallel. A general distinction between studies investigating residential settings and occupational settings was made for content purposes.

### 2.5. Collating the Data

Based on the study findings, implications for research are formulated [22], meaning that research gaps are identified and the need for future research is highlighted. This scoping review step is done within the Section 4.

## 3. Results

### 3.1. Study Selection

Via the comprehensive literature search, nine studies were identified that met the inclusion criteria, of which six were identified via database searches and three via other searches. For the initial database searches (from 2 February, 2016), the proportion of agreement between the two reviewers for title-abstract screening was 0.99 and Cohen’s Kappa 0.40 (fair), and for full text screening 0.90 and 0.80 (substantial), respectively.

**Figure 1:**
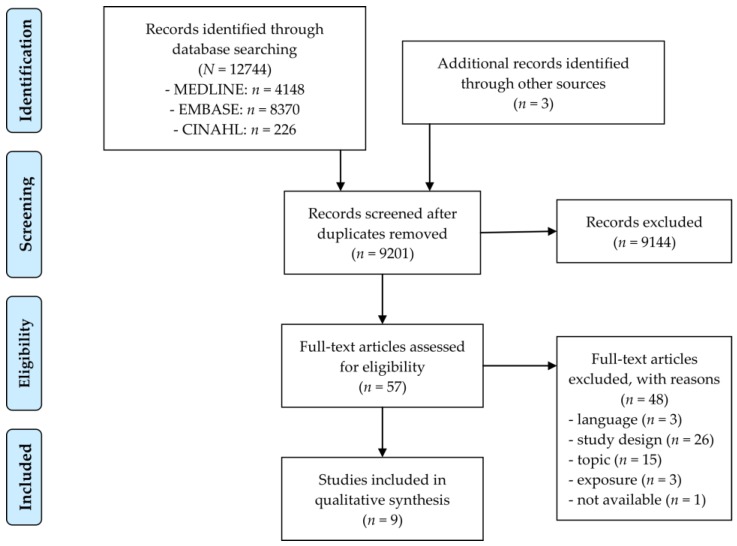
Study selection process.

Figure 1 illustrates the process of study selection in detail, covering the number of search hits retrieved with database searches (*n* = 12,744) and all other searches (*n* = 3), the number of screened titles/abstracts (*n* = 9201) and full texts (*n* = 57), and the number of finally included studies (*n* = 9). The number of hits from all other searches (*n* = 3) comprises only relevant ones, as it is not possible to quantify the actual number of references screened (e.g., on homepages).

### 3.2. Study Characteristics

Of the nine relevant studies, four were cross-sectional studies [23,24,25,26], two content analyses [27,28], two exposure studies (with a secondary literature analysis) [29,30], and one a case series [31]. Only one of the four cross-sectional studies masked the study purpose [23]. Eight articles, except one [31], were published in peer-reviewed journals. All publications were written in English. From one study, two articles were published, each with a focus on different exposures [29,30]. These two studies were based on measured exposure levels. However, outcome data were not evaluated, but calculated with a risk assessment program. So in fact, these studies are rather exposure studies than “epidemiological” exposure-effect studies. We nevertheless decided to keep these studies, as health outcomes were taken into account. All but one study (from Thailand) were conducted in Europe—two each in Denmark, Finland, and United Kingdom, and one each in Sweden and Germany. Six articles took place in occupational settings [23,26,27,29,30,31] and two in residential settings [24,25]. One study did not differentiate between occupational and residential settings [28]. Of the occupational setting-studies, four occurred in biomass-fired power plants investigating endotoxin exposure [23], multiple exposure to gases [29] or metals [30], or working in this specific setting as a very general formulated exposure [26], and two in biogas plants examining the effects of hydrogen sulfide intoxication [27,31]. Outcomes of interest were mainly respiratory symptoms, allergic reactions, skin complaints, but also central nervous system disorders, general health, and fatalities. Two studies analyzed the impact of living near biomass-fired power plants on residents` odor annoyance as well as different health symptoms and diseases [24,25]. One content analysis compared the global absolute and relative fatality rate of energy production and distribution of biomass with other conventional as well as renewable energy forms [28]. Table 2 details the characteristics of each included study.

### 3.3. Study Results and Assessment of Study Methods

#### 3.3.1. Occupational Setting—Biomass-Fired Power Plants

A cross-sectional study from Denmark investigated the association of endotoxin exposure in a biomass power plant with different health outcomes if plant employees and found that it is statistically significantly associated with chronic bronchitis and wheezing, but not with asthma, Organic Dust Toxic Syndrome, hay fever, allergy, and atopy [23]. With data from the same power plant and population, Schlunssen et al. observed that working in a biomass-fired power plant is not statistically significantly associated with an increased risk for respiratory diseases compared to working in a conventional power plant, but the exposures to endotoxins as well as fungi is associated with respiratory symptoms (e.g., asthma, nose symptoms) [26].

The studies of Basinas et al. and Schlunssen et al. were based on the same data set, thus the methodological quality-evaluation is almost identical. Nearly all categories were evaluated as having a high methodological quality. Reporting was adequate in both studies. No selection bias was apparent (census sampling, high response). The comparison groups were determined to be eligible. The exposure rates were measured with job-exposure matrices for each worker based on diary recordings for personal working tasks and working areas from one week and based on substance levels obtained from stationary dust samples. For logistic regression analyses, important confounders were considered. A high methodological quality in regard to ethical topics was found in both articles. But as the data were evaluated in 2003, it is unclear whether the study findings are generalizable. The only different judgment concerns the information bias of the outcome parameters. In the article of Basinas et al., the diagnosis of atopy was determined with valid diagnostic criteria (high methodological quality), but the other health symptoms were assigned with a low methodological quality-rating since these were studied with subjective single questions. The outcome parameters investigated by Schlunssen et al. were diagnosed with detailed clinical definitions and thus were of high methodological quality.

Two articles authored by Jumpponen et al. examined health effects resulting from multiple exposure to gases and metals in biomass-fired power plants [29,30]. It was found that the measured gas exposure magnitude might comprise the risk of upper respiratory tract irritation and central nervous system disorders. The measured multiple exposure to metals might increase the risk of cancer, lower respiratory tract irritation, upper respiratory tract irritation, and central nervous system disorders. With respect to multiple exposure to gases, upper respiratory tract irritation might be explained by the combined effects of sulfur dioxide, nitric oxide, nitrogen dioxide, ammonia, and hydrogen sulfide, while central nervous system disorders might stem from the combined effects of carbon monoxide and hydrogen sulfide. Regarding multiple exposure to metals, an elevated cancer risk might be due to the combined effects of arsenic, beryllium, cadmium, and lead; central nervous system disorders might be explained by the combined effects of manganese, lead, and selenium; lower respiratory tract irritation by the combined effects of beryllium, cadmium, manganese, and selenium; and upper respiratory tract irritation by the combined effects of aluminium, arsenic, and selenium. According to the study findings, these increased health risks are caused by combined effects of various substances, not by the effect of a single substance.

Both studies were appraised as a content analysis and exhibited the same methodological assessment results. Reporting was of low methodological quality since specifications about the outcome parameters and the risk assessment program used were missing. The eligibility of the comparison group and selection bias was unclear due to a lack of appropriate information about the risk assessment program used (or more specifically: the underlying limit values of the risk assessment program). The exposure levels of gases and metals were measured with air samples and thus judged with a high methodological quality. It is unclear if an information bias regarding the outcome parameters is apparent, since the outcome definitions used within the risk assessment program were not outlined. It remains unclear if confounders were considered within the analyses of the risk assessment program. No statement of conflict of interest was available. Funding sources were given and seem to be of high methodological quality. Study findings seem to be generalizable, since different types of biomass plants were included and all biomass power plants in Finland were asked to participate.

#### 3.3.2. Occupational Setting—Biogas Plants

Two studies reported immediate fatalities and other health symptoms (e.g., nausea, unconsciousness) occurring among power plant workers and other on-site employees after accidental exposure to hydrogen sulfide in biogas plants [27,31].

The case series lacked important information for a better scientific evaluation [31]. The study design itself evokes a selection bias (convenience sampling, no control group). No details on the hydrogen sulfide level during the incident were given, but the accident as such was analyzed in detail. The variables “fatality” and “unconsciousness” are easy to establish, so there is a high methodological quality of the category “information bias” regarding these outcome parameters. Ethical aspects were not issued (and are not expected in such reports). Generalizability is not given, since this is the description of a single case.

The content analysis did not specify its objectives, but overall reporting was sufficient [27]. The category “selection bias” is judged as being of high methodological quality as all medical files in a defined period were surveyed. The category “information bias of the exposure” was of low methodological quality as the content analysis did not provide information about the exposure level of hydrogen sulfide during the incident, but an in-depth description of the accident. There was a high methodological quality of the information bias in regard to the outcome parameters as these were assessed and documented by medical professionals. The study did not make a statement about conflict of interest or funding. It is uncertain if such an accident is transferable to today`s technical and organizational standards of biogas plants.

#### 3.3.3. Residential Setting

One cross-sectional study reported that the odorous air pollution emanating from a biofuel facility (caused by organic substances like terpenes, smoke, and dust) did not have a direct impact on odor annoyance and health symptoms among residents living near to it, but the association was mediated by the perception of the pollution as well as a health risk perception [24]. The reporting of the article was of high methodological quality. The study population was selected randomly and the response was high (65%), thus the category “selection bias” was of high methodological quality. The comparison group is judged as being not eligible as the classification of odorous air pollution is based on the distance to the odor source (estimated by postal codes). For the same reason, an information bias regarding the exposure was identified (low methodological quality). It remains unclear how the outcome parameters were operationalized and if confounders were considered. The ethical aspects “conflict of interest” and “funding” are of high methodological quality. No information was given about the approval of an ethics committee. Only one biofuel plant was examined, thus generalizability is questionable.

In another cross-sectional study from Thailand, residents living in the vicinity of two small biomass power plants showed an increased risk for suffering from respiratory diseases (asthma, COPD), allergy, and certain symptoms (e.g., itching/rash, eye irritation, cough, stuffy nose, allergic symptoms, sore throat, and difficult breathing) [25]. The reporting of objectives, methods, and results were adequate, but information on sampling, response, operationalization of the outcome parameters, confounders, conflict of interest, and ethics committee approval were missing. An information bias in regard to the exposure seems to be existent (low methodological quality), as the exposure was determined by the distance between the residence and the biomass plant. Generalizability is of low methodological quality due to the restricted setting.

#### 3.3.4. Occupational and Residential Setting

Sovacool et al. counted 97 fatalities worldwide occurring from 1874 till 2014 through the energy production from and distribution of biomass (comprising wood, agricultural residues, cellulosic energy crops, waste, and biogas) [28]. The normalized risk per terawatt hour was 0.0164. The absolute number of fatalities was smaller compared to fossil fuels and hydroelectricity, and equal to other forms of renewables. The relative risk was fourth highest among all other energy forms, and only lower than wind energy, hydroelectricity, and solar power. Reporting of the study was very extensive. It was unclear if a selection bias was available. On the one hand, the search was very comprehensive and comprised only published documents, but on the other hand no information on search sources used and number of persons involved in searches were given and only documents written in English were taken into account. The comparison groups (other forms of energy) seem to be eligible for the scope of this scoping review. The exposure variable was clearly defined. There is no information bias regarding the outcome parameter as fatality information can easily be determined (high methodological quality), but confounders were not integrated in the data analysis. Ethical issues (conflict of interest, funding) were not issued by the authors. Study results seem to be generalizable as a worldwide population and setting was incorporated in the analysis.

Table 3 outlines the study results. Statistically significant results are highlighted in bold. The study findings were not summarized meta-analytically due to the low number of included studies and their heterogeneous nature.

Table 4 illustrates the methodological assessment of each study. A high methodological quality of a category is presented with a plus-symbol (+), and a low methodological quality with a minus-symbol (−). Unclear assessments are designated as such.

## 4. Discussion

### 4.1. Summary of the Body of Evidence

Nine studies were retrieved that researched the health impacts resulting from conversion and combustion of biomass for electricity generation. Included studies found that the local exposure to endotoxins and fungi is associated with respiratory symptoms and diseases (e.g., chronic bronchitis, wheezing) [23,26]. Further, multiple exposure to different gases (e.g., sulfur dioxide, nitric oxide, nitrogen dioxide, ammonia, hydrogen sulfide, carbon monoxide) may explain an elevated risk of respiratory and neurotoxic effects [29], and multiple exposure to metals (e.g., arsenic, beryllium, cadmium, lead, manganese, selenium, aluminium) an elevated risk of carcinogen, neurotoxic, and respiratory effects [30]. In biogas plants, there is a risk of hydrogen sulfide intoxication in case of an accidental leakage of the gas resulting in fatalities or severe symptoms among workers [27,31]. One study showed that living near a biomass power plant increased the risk of respiratory disorders and skin complaints [25]. Another study found that the odorous air pollution emanating from biofuel facilities did not directly influence annoyance and health symptoms among residents, but the perception of this pollution and the evaluation of a health risk by this pollution did [24]. One content analysis outlined that the relative risk for a fatality through biomass production and distribution is forth highest among many other forms of energy [28].

Although not considered in this scoping review, earlier life cycle phases of biomass may pose a risk for workers’ health. During the production of the feedstock, hazards are considered to be similar to those of agriculture and forestry [32]. Thermal processing is associated with exposure to different risky substances (e.g., carcinogens, gases, carbon monoxide, sulfur oxides, lead, and volatile organic compounds) [32]. During the storage of biomass there is an elevated risk for explosion, fire, and local air pollution [32].

Focusing on the combustion phase of biomass, Rohr et al. noticed that issues concerning occupational safety and health among workers in bioenergy plants are influenced by the properties and characteristics of the biomass fuels itself, the plant design, and also by the fuel processing, handling, and storage [12]. Exposed workers may be fuel-handling personnel who transport, store, and prepare biomass at the site, cleaners who remove dust deposits from the plant, maintenance engineers, outage contractors who repair plant items during shutdown periods, ash-handling personnel, and other plant personnel [12].

The evaluation of potential occupational exposures in bioenergy plants is complicated due to the variety of fuel types and facility designs as well as missing scientific monitoring data, making comparisons with similar industries the more challenging [12]. Exposure studies measuring levels of substances occurring in bioenergy plants are rare. A team of authors measured exposure levels of various substances in Danish bioenergy plants [33,34,35,36]. Levels of dustiness varied between different biomasses (e.g., straw, wood pellets, wood chips, wood briquettes) [34], but, in general, exposure levels of endotoxin, actinomycetes, bacteria, and fungi [35] as well as of particulate matter [36] seemed to be high. Increased levels of Interleukin 1β was found among plant workers in their exhaled breath condensate, pointing to a sub-chronic and chronic inflammation of the respiratory tract [33]. Another exposure study was conducted in three biomass heat and power plants in Finland [37]. During the processing of biomass, workers were exposed to high levels of actinobacteria, bacterial endotoxins, and fungi, as well as to organic dust and volatile organic compounds. During operation, there was an exposure to endotoxins, actinobacteria, and fungi which exceeded the limit values proposed by the Finnish Institute of Occupational Health. There are no studies on specific exposure patterns occurring exclusively in bioenergy plants. It may be possible that even if recommended threshold limits of all substances are complied with, there are still health impacts due to the combined effects of the various substances. Jumpponen et al. revealed such potential additive interactions [29,30].

The risk assessment studies by Jumpponen et al. [29,30] were included in this scoping review as health outcome results were estimated. For this purpose, measured exposure values were entered into a risk assessment program (called MIXIE), which computed the risk for various diseases. The papers provided no information on the underlying limit values used within the risk assessment program. Generalization of the study findings was rated to be of low risk of bias, since all Finnish biomass power plants were asked to participate. Nevertheless, international comparability could be questioned, as it is expected that other countries have different occupational exposure levels.

The Jumponnen et al.-study from 2013 measured levels of different substances (e.g., carbon monoxide or polycyclic aromatic hydrocarbons (PAHs)) [29]. Carbon monoxide is known for its toxicity, potentially leading to acute carbon monoxide poisoning [38]. Typically, chronic exposure to carbon monoxide leads to a reactive polyglobuly due to long-term hypoxia [39]. In such cases, affected persons develop a relative tolerance against acute carbon monoxide intoxication. Thus, chronic effects of the intoxication with carbon monoxide are controversial [39]. According to Jumponnen et al. combined exposure to carbon monoxide and hydrogen sulfide may lead to central nervous system disorders among workers in bioenergy plants [29]. PAHs are classified as dangerous substances due to their cancerogenity [40]. In the Jumponnen-study, air concentrations of PAHs were less than 7% of the Finnish Occupational Exposure Limits (of benzo(a)pyrene) for ash removal and maintenance tasks [29]. The study did not assess the health impacts of PAHs among bioenergy plant workers.

In the 2014-study of Jumponnen et al., levels of aluminium, manganese, and lead were high and partly exceeded Finnish occupational exposure limits [30]. High workplace concentrations of aluminium are known to eventually cause lung fibrosis, called aluminosis, as well as having central nervous system effects [41]. Long-term exposure to manganese may lead to chronic manganism, a parkinsonism-like disorder, and to pneumonia [42]. Lead can affect the hematopoietic system (erythrocyte production, hemoglobin synthesis), and induce neurotoxic effects (e.g., central nervous system disorders, disorders of peripheral motor and smooth muscular function) [43].

Preventive measures to avoid health impacts in biomass power facilities comprise technology controls and measures (e.g., isolation of the fuel reception hall, crushers, and screens; enclosure of conveyors; control rooms for supervision of unloading fuel trucks; automated fuel sampling; automatic cleaning systems for fuel trucks), worker training on the correct handling of biomass and resulting ashes, improvement of the quality of fuels, and protective clothing (e.g., hoods) and respirators [12,37]. Technical solutions should be preferred to protective clothing and respirators [37]. Some of these measures (especially of the post-combustion phase) are well known from the fossil fuel industry [32]. Furthermore, immission control legislations aim to prevent, reduce, and eliminate pollution arising from industrial activities. For example, the Industrial Emissions Directive of the European Parliament and the Council of the European Union sets out emission limit values for biomass combustion plants (expressed as milligram per standard cubic meters (mg/Nm^3^)) according to the total rated thermal input (expressed as megawatt (MW)) concerning sulfur dioxide (50–100 MW: 200 mg/Nm^3^, 100–300 MW: 200 mg/Nm^3^, >300 MW: 200 mg/Nm^3^), nitrogen dioxide (50–100 MW: 300 mg/Nm^3^, 100–300 MW: 250 mg/Nm^3^, >300 MW: 200 mg/Nm^3^), and dust (50–100 MW: 30 mg/Nm^3^, 100–300 MW: 20 mg/Nm^3^, >300 MW: 20 mg/Nm^3^) [44]. Since emissions from biomass combustion plants may have an impact on human health (as outlined above) such legislations are necessary, even though their effectiveness have not been shown yet.

Included studies about biogas plants mentioned health effects of hydrogen sulfide. The gas is only detectable by its smell at very low concentrations (around 0.02 part per million (ppm)), with a recommended workplace concentration of 10 ppm [45]. Measures to prevent intoxication with hydrogen sulfide in biogas plants are amongst others appropriate education of staff, risk assessment at the facility level, and (fixed and portable) gas detectors [45]. In most cases of intoxication, patients die within seconds or minutes, or their cardiac contractility and cortical and medullary functions may be affected [46]. No studies about other risk factors associated with working in biogas plants were identified within this scoping review. But further impacts may be expected, such as odor annoyance, intensified road traffic, residues of pesticides and veterinary drugs in agricultural fertilizers, or competition with food production [47]. One exposure study of two biogas plants in Italy using different feedstocks (e.g., silage, corn cobs, fruit/vegetable waste, cattle manure) found varying mean levels of microbes (Global Index of Microbial Contamination: 876 (plant 1), 16,154 (plant 2)), and low mean levels of particulate matter 10 (21.42 µg/m^3^ (plant 1), 36.35 µg/m^3^ (plant 2)) and endotoxins (3.08 endotoxin units/m^3^ (plant 1), 8.33 endotoxin units/m^3^ (plant 2)) compared to recommended exposure guidelines [48]. Since different factors (e.g., nature of biomass, storage conditions, plant characteristics) influence exposure levels of such substances, the study authors indicate that it is important to evaluate the health risk occurring in biogas plants on an individual facility level. With regard to environmental legislation, exemplary in Germany, biogas plants that produce more than 1.2 million cubic meters biogas per year are subject to approval (regulated by the Fourth Ordinance Implementing the Federal Immission Control Act) [49] and even bigger biogas plants are subject to the Major Accidents Ordinance [50]. 

In relation to health impacts of energy generation in the occupational context, it was found that energy generation with biomass is comparable with the fossil fuel industry and has more negative implications on workers than the wind and solar sector (during the extraction, generation, and distribution phase) [12,32,51]. 

The results of the scoping review mainly address health effects of biomass-use for electricity generation on the individual´s level. It is also important to consider a public health perspective by comparing the impact of this form of energy generation with other forms like fossils fuels, or other renewables. Fossil fuels have the most threatening public health consequences. Besides the fact that utilizing fossil fuels leads to global warming, resulting in climate change and negative health impacts, it further jeopardizes human health by emitting substances like nitrogen oxides, sulfur dioxide, heavy metals, and organic substances on a large scale [3,52]. Biofuels reduce greenhouse gas emissions relative to fossil fuels and increase climate benefits (e.g., land use changes, fossil fuel inputs), but the latter only on a marginal scale [53]. It is assumed that the resulting chronic mortality of biomass electricity generation is less than 20% of the rate of lignite technologies [3]. Fatality rates of biogas facilities are much lower than those of fossil fuels, and equal to those of hydro and nuclear power [54]. Due to their decentralization, their risk for catastrophic events is minimal, in contrast to the maximum consequences of fossil fuels, hydropower, or nuclear power [54]. Despite this, the advantages for public health of biomass energy generation compared to those of fossil fuels seem to be minimal, and it appears that its whole life cycle phase is still in need of some optimization through, amongst others, developing efficient crops and production technologies, or using waste material and residues rather than specially planted biofuels [53].

### 4.2. Methodological Aspects of the Studies Included in the Scoping Review

The results of the methodological assessment of the included studies varied a lot. Two cross-sectional studies from the occupational setting were of high methodological quality of almost all categories [23,26]. In two studies from the occupational setting [29,30] and one study from the residential setting [25] the assessment of most categories was unclear due to missing information. One paper was mainly of low methodological quality [31]. The within quality assessment of three studies was heterogeneous [24,27,28].

The reporting quality of most studies was high. The sampling process used in three of five epidemiological studies (census or random selection) and the response rate in three of the four cross-sectional studies was of high methodological quality (>50%). The occurrence of a selection bias was negated or unclear for four studies, respectively, and affirmed for one study. Heterogeneous assessments were obtained for the “information bias of the exposure” (low or high methodological quality-ratings) and for the “information bias of the outcome” (high or unclear methodological quality-ratings). Only two studies considered confounders in their data analysis. Most studies did not report a conflict of interest-statement, but declared the funding sources. An ethics committee approved the study conduct of only two studies. Generalization was of unclear or low methodological quality in most studies.

### 4.3. Need for Future Research

Regarding the occupational setting, there are few epidemiological studies about health impacts occurring in biomass-fired power plants and biogas plants. Comprehensive international research on exposure levels of all substances that are present in biomass-fired power plants is missing. The few exposure studies indicate that exposure levels of health endangering substances like endotoxins or fungi are rather high and sometimes exceed recommended occupational exposure levels. It is further unclear if new exposures or exposure patterns are existent in bioenergy plants (compared to other occupational settings). Current scientific knowledge is mostly derived from research of other industries where similar substances appear. As the sector of energy production by biomass-use is rapidly growing; this research gap is of concern [55].

Only two cross-sectional studies in residential settings were retrieved, which showed the occurrence of some negative health effects of biomass power plants among residents living near those facilities (e.g., odor annoyance, respiratory complaints).

Thus, both, health effects resulting from biomass-use for electricity generation on humans in occupational settings as well as in residential settings, need to be studied comprehensively. In a first step, the levels of exposure of all substances that are present in biomass power plants as well as specific exposure patterns should be determined in exposure studies and biomonitoring studies. These levels should be compared to threshold values and dose-response relationships that are already established for several substances (e.g., beryllium, lead, cadmium). Such reference points may be national occupational exposure limits (e.g., various Technical Rules for Hazardous Substances of the German Committee on Hazardous Substances [56]) or threshold values that were determined in scientific papers. If these comparisons raise concern for health consequences, appropriate high-quality observational studies, should be conducted in a second step. Ideally, such observational studies should select participants randomly or as a census, obtain a high response, have an eligible control group (occupational setting: general working population, residential setting: general population not living in the vicinity of bioenergy plants), determine all substances of relevance, measure exposures and outcomes with valid and reliable instruments, consider appropriate confounders in data analyses, have a follow-up investigation, take ethical aspects into account, and ensure generalization of the study results.

### 4.4. Strengths and Weaknesses of the Scoping Review

This is the first comprehensive scientific overview that outlines the topic of electricity generation through biomass-use and resulting health impacts on humans in occupational settings as well as in residential settings. The literature search was designed to be sensitive and broad. The review process was rigorous, systematic, and transparent, and review steps were verified by a second independent reviewer.

Assessing the methods of included studies is not mandatory in scoping reviews, but was done as a part of this work to show methodological aspects of each paper and to stimulate a critical interpretation of the related study findings among readers. Determination of the methodological quality was carried out by one reviewer based on the evaluation of methodological strengths and weaknesses of each study, which was done in duplicate. Since different study designs were retrieved, no single, validated critical appraisal tool could be used.

Due to the methodological nature of a scoping review presenting the whole body of evidence on a broad research topic and screening for potential research needs, the study results were not summarized in a meta-analytical manner. In addition, this would not have been possible due to the low number and heterogeneity of included studies. As only studies written in German and English were included, a language bias may exist.

## 5. Conclusions

The scientific literature on health impacts resulting from conversion and combustion of biomass for power generation is sparse, both in residential and in occupational settings. To date, statements about health impacts of the use of biomass for electricity generation are mainly derived from other industries with similar exposures. Two cross-sectional studies of high methodological quality identified within this scoping review suggest that there is an association between exposure to endotoxins and fungi and respiratory disorders among workers in biomass-fired power plants. Two exposure studies, which extrapolated health outcomes based on measured concentrations, found that exposure to multiple gases may lead to an increased risk of respiratory and neurotoxic diseases, and exposure to multiple metals might comprise the risk of cancer, neurologic, and respiratory diseases among workers in biomass-fired power plants. There exists a risk for the occurrence of severe symptoms and fatalities in case of an accidental hydrogen sulfide leakage in biogas plants. Living near biomass power plants (and the related exposure to odorous air pollution) resulted in an increased risk for several symptoms and odor annoyance in one cross-sectional study of mainly uncertain methodological quality, and this association may be mediated by the perception about this air pollution or the evaluation of resulting health risks according to a cross-sectional study of mostly high methodological quality. The methodological quality of included studies varied a lot, from studies with mainly high methodological quality-ratings, to studies with mainly unclear or low methodological quality-assessments. To draw definitive implications on health impacts of electricity generation by biomass –use, the levels of exposure of all substances that are present in biomass power plants should first be determined and compared to established threshold limits in future studies. If these levels raise concerns for health impairments, appropriate high-quality observational studies should be conducted in a second step, which especially attach importance to appropriate sampling (census, or random selection), eligible control groups, valid and reliable measuring methods of the exposure and outcome, and consideration of relevant confounders.

## Figures and Tables

**Table 1:** Inclusion and exclusion criteria.

Category	Inclusion Criteria	Exclusion Criteria
Population	- humans living in the vicinity of bioenergy plants (residential setting) working in bioenergy plants (occupational setting)	- animals
Exposure	- biomass-use for electricity generation, comprising the following life cycle phases of biomass-use for electricity generation: conversion of biomass to an energy carrier that will be burned later (e.g., biogas plants) combustion of biomass for electricity generation (isolated or in combination with biomass-use for heat generation) (e.g., biomass-fired power plants)	- the following life cycle phases of biomass-use for electricity generation: harvesting, collecting, and providing biomass preparation, storage, and transport of biomass - biomass-use for heat or fuel generation only
Outcome	- effects on human health (complaints, diseases) - effects on human safety (injuries, fatalities)	- physiological parameters - surrogate markers
Study design	- observational study (cohort study, case-control study, cross-sectional study, case series, case report, ecological study) - intervention study (randomized controlled trial, non-randomized controlled trial, before-after study) - qualitative study (interview, focus group discussion) - experimental study - review with a systematic review approach - content analysis	- review without a systematic review approach (narrative review) - subjective study type (editorial, commentary, expert opinion) - animal study - monitoring study - exposure study - only abstract available

**Table 2:** Study characteristics of included studies.

**Studies from Occupational Settings**							
**Study**	**Study Design**	**Period**	**Country**	**Setting**	**Population**	**Exposure (Operationalization)**	**Outcome Parameter (Operationalization)**
Basinas et al., 2012 * [23] (English)	Cross-sectional study ^1^	2003	Denmark	(Straw, wood) power plant	Energy plant employees: *N* = 232 - straw, *n* = 94 - wood, *n* = 138 Female: 4% Mean age: 47.6 yr. Response: 74/75%	Endotoxin exposure: Current personal endotoxin exposure estimated by means of quantitative Job-Exposure Matrices (estimated based on information on the time spent on each working task or area from one week exposure diaries and endotoxin levels obtained from 181 stationary dust samples collected in all working areas) 3 exposure groups: - low: <50 EU/m^3^ - medium: 50–1000 EU/m^3^ - high: >1000 EU/m^3^ Median estimated average endotoxin exposure: 0.01–294 EU/m^3^	Asthma, chronic bronchitis, hay fever, allergy, organic dust toxic syndrome, wheezing: Subjective questions about each disease/complaint Atopy: Elevated serum immunoglobulin E concentration, skin prick test
HSE, 2015 [31] (English)	Case series	June 2009	United Kingdom	Biogas plant	Farm worker: *n* = 6 Female, age: NS Response: NA	Hydrogen sulfide exposure: - during maintenance tasks - duration: few seconds - exposure level: NS	General health, unconsciousness, fatality: Descriptive report of the Health and Safety Executive (HSE)
Jumpponen et al., 2013 * [29] (English)	Exposure study (with a secondary literature analysis)	2010	Finland	Biomass-fired power plants: - pellets: *n* = 2 - wood: *n* = 3 - peat: *n* = 2 - recycled fuels: *n* = 1	Energy plant employees: *N* = 35 - maintenance, *n* = 17 - ash removal, *n* = 18 Female: 0% Mean age: 37 yr. Response: NS	Multiple exposure to gases: Air samples from stationary sampling points during ash removal and maintenance tasks using TSI and X-am 7000 (Dräger) gas monitors Exposure values, on which risk assessment was based (see Table 3): (*maximum concentrations of each gas*) - carbon monoxide: 46 ppm - nitric oxide: 30 ppm - ammonia: 11 ppm - sulfur dioxide: 17 ppm - nitrogen dioxide: 0.5 ppm - hydrogen sulfide: 2 ppm	Upper respiratory tract irritation, central nervous system disorders: Risk assessment program (“Mixie computer-based tool”)
**Studies from Occupational Settings**							
**Study**	**Study Design**	**Period**	**Country**	**Setting**	**Population**	**Exposure (Operationalization)**	**Outcome Parameter (Operationalization)**
Jumpponen et al., 2014 * [30] (English)	Exposure study (with a secondary literature analysis)	2010	Finland	Biomass-fired power plants: - pellets: *n* = 2 - wood: *n* = 3 - peat: *n* = 2 - recycled fuels: *n* = 1	Energy plant employees: *N* = 35 - maintenance, *n* = 17 - ash removal, *n* = 18 Female: 0% Mean age: 37 yr. Response: NS	Multiple exposure to metals: Air samples from breathing zones of the workers or from stationary sampling points during ash removal and maintenance tasks using an IOM sampler - sampling period: 53–464 min Exposure values, on which risk assessment was based (see Table 3): (*mean concentrations of each metal*) - aluminium: ash removal: 2.0 mg/m^3^, maintenance: 1.9 mg/m^3^ - arsenic: ash removal: 0.007 mg/m^3^, maintenance: 0.003 mg/m^3^ - lead: ash removal: 0.07 mg/m^3^, maintenance: 0.02 mg/m^3^ - cadmium: ash removal: 0.003 mg/m^3^, maintenance: 0.0007 mg/m^3^ - manganese: ash removal: 0.7 mg/m^3^, maintenance: 0.4 mg/m^3^ - selene: ash removal: 0.002 mg/m^3^, maintenance: 0.0001 mg/m^3^ - beryllium: ash removal: 0.0001 mg/m^3^, maintenance: 0.0001 mg/m^3^	Upper and lower respiratory tract irritation, central nervous system disorders, cancer: Risk assessment program (“Mixie computer-based tool”)
Oesterhelweg & Püschel, 2008 * [27] (English)	Content analysis	Search period: 1980–2005	Germany	Biogas plant	Employees: *N* = 4 - power plant, *n* = 3 - truck driver, *n* = 1 Female: 25% Age range: 28–50 yr. Response: NA	Hydrogen sulfide exposure: Reconstruction of the technical analysis executed at the scene by the police and fire department - exposure level: NS	Fatality, general health: Autopsy files of the Department of Legal Medicine, Hamburg (Autopsy performed within 36 h after the incident)
**Studies from Occupational Settings**							
**Study**	**Study Design**	**Period**	**Country**	**Setting**	**Population**	**Exposure (Operationalization)**	**Outcome Parameter (Operationalization)**
Schlunssen et al., 2011 * [26] (English)	Cross-sectional study	2003	Denmark	(Straw, wood) power plant	Energy plant employees: *N* = 232 - straw power plant, *n* = 94 - wood power plant, *n* = 138 - control group: *n* = 107 Female: ca. 4% Mean age: 45.9–48.1 yr. Response (exposure groups): 74/75%	Working in a (straw, wood) power plant: (Exposure to dust, endotoxins, fungi, Aspergillus fumigatus) Operationalization: (a) type of power plant:- exposure groups: straw power plant, wood power plant - control group: conventional power plant (b) personal current average exposure levels of dust, endotoxin, cultivable fungi: - estimated from stationary work area measurements and information on time spent on each work task or in each work area (recorded in a diary) - dust: extracted with Teflon filters- endotoxin: measured with kinetic Limulus Amboecyte Lysate test - fungi: measured with Biap slit-to-agar sampler (sampling time: 1 min, flow: 106 L/min) Exposure values (*median*, *range*): - dust (mg/m^3^): wood: 0.03 (0.01–0.1), straw: 0.13 (0.02–0.33) - endotoxin (EU/m^3^): wood: 1.7 (0.01–6.5), straw: 74 (1.5–294) - cultivable fungi (cfu/m^3^): wood: 1.03x10^3^ (363–5.01 × 10^3^), straw: 5.28 × 10^3^ (119–1.84 × 10^4^) - Aspergillus fumigatus (cfu/m^3^): wood: 241 (0–1.32 × 10^3^), straw: 1.04 × 10^3^ (6.2–2.78 × 10^3^)	Respiratory health symptoms (e.g., asthma, rhinitis, chronic bronchitis): Detailed outcome definitions (see Schlunssen et al., 2011 [26])
**Studies from Residential Settings**							
**Study**	**Study Design**	**Period**	**Country**	**Setting**	**Population**	**Exposure**	**Outcome Parameter**
Claeson et al., 2013 * [24] (English)	Cross-sectional study	May (year: NS)	Sweden	Biofuel facility for power and heat generation	Residents: *n* = 722 Female: 57.6% Age distribution: - 18–29 yr.: 18.3% - 30–44 yr.: 32.3% - 45–64 yr.: 36.0% - >64 yr.: 13.4% Response: 65%	Odorous air pollution: Organic substances (terpenes, smoke, dust) Exposure groups: - estimated according to post codes - low: post code 1124 - medium: post codes 1231 and 1251 - high: post code 1241	Odor annoyance: No information on measurement methods available Health symptoms (fatigue, feeling heavy headed, headache, nausea, dizziness, attentional difficulties, eye itching/burning/irritation, nose irritation/congestion/running, hoarseness/dry throat, coughing, face skin dryness/redness): No information on measurement methods available
Juntarawijit, 2013 * [25] (English)	Cross-sectional study	NS	Thailand	Biomass-fired power plants: - steam turbine: *n* = 1 - gasification: *n* = 1	Residents: - chronic diseases, *n* = 1254 - health symptoms, *n* = 392 Female, age, response: NS	Living near biomass power plants: - measured in distances living away from the biomass power plant (self-assessed by the residents) Exposure group: - I: 0–0.5 km living away from plant - II: 0.5–1.0 km living away from plant Control group: - > 1 km living away from plant	Chronic diseases (allergy, asthma, heart disease, COPD, tuberculosis, cancer): No information on measurement methods available Health symptoms (itching/rash, eye irritation, cough, stuffy nose, allergic symptoms, sore throat, difficulty breathing): No information on measurement methods available
**Studies from Occupational and Residential Settings**							
**Study**	**Study Design**	**Period**	**Country**	**Setting**	**Population**	**Exposure**	**Outcome Parameter**
Sovacool et al., 2015* [28] (English)	Content analysis	Search period: 1874–2014 Search duration: 6 months	United Kingdom	Biomass facilities for power generation and distribution	Humans	Biomass (wood, agricultural residues, cellulosic energy crops, waste, biogas): Inclusion criteria: energy production and distribution Exclusion criteria: energy consumption or downstream pollution and externalities	Fatalities: No information on measurement methods available

cfu: colony-forming units, COPD: chronic obstructive pulmonary disease, EU: endotoxin units, km: kilometer, L: litre, mg: milligram, min: minute, m^3^: cubic metres, *n*/*N*: sample size, NA: not applicable, NS: not specified, ppm: parts per million, yr.: years. * peer-reviewed publication. ^1^ masking of study purpose.

**Table 3:** Study results of included studies.

Studies from Occupational Settings			
Study	Exposure	Results	Influencing Factors
Basinas et al., 2012 [23]	Endotoxin exposure in a straw/wood power plant	**Association “endotoxin exposure and …”:** (*logistic regression*, *adjusted for atopic predisposition*, *gender*, *smoking habit*, *age*, *farming during childhood*, *control group: lowest exposure*) **Chronic bronchitis:** - medium exposure: **OR = 11.05** (95% CI: 1.27–96.35) - high exposure: OR = 8.44 (95% CI: 0.49–145.09) **Wheezing:** - medium exposure: OR = 1.78 (95% CI: 0.62–5.09) - high exposure: **OR = 5.09** (95% CI: 1.28–20.24) **Asthma:** - medium exposure: OR = 1.32 (95% CI: 0.50–5.49) - high exposure: OR = 3.60 (95% CI: 0.93–13.94) **Organic Dust Toxic Syndrome:** - medium exposure: OR = 2.59 (95% CI: 0.95–7.05) - high exposure: OR = 0.77 (95% CI: 0.09–6.82) **Hay fever:** - medium exposure: OR = 0.68 (95% CI: 0.21–2.24) - high exposure: OR = 1.61 (95% CI: 0.36–7.08) **Allergy:** - medium exposure: OR = 0.34 (95% CI: 0.09–1.30) - high exposure: OR = 0.55 (95% CI: 0.06–5.07) **Atopy:** - medium exposure: OR = 0.83 (95% CI: 0.25–2.82) - high exposure: OR = 1.36 (95% CI: 0.24–7.79)	/
HSE, 2015 [31]	Hydrogen sulfide gas exposure in a biogas plant	**Descriptive analysis:** - unconsciousness: *n* = 2 - fatality: *n* = 1 - further health effects (not further defined): *n* = 4	/
Jumpponen et al., 2013 [29]	Multiple exposure to gases in biomass-fired power plants	**Effects of multiple exposure to gases:** (*only power plants and tasks reported that cause effects*, *percentages refer to Finnish Occupational Exposure Limits; mean* ± SD) **Upper respiratory tract irritation:** (*caused by combined effects of* *sulfur dioxide*, *nitric oxide*, *nitrogen dioxide*, *ammonia*, *and hydrogen sulfide*) - peat-firing (maintenance): 350 ± 57% - recycled fuel-firing (ash removal): 150 ± 59% - wood-firing (ash removal): 36 ± 15% - pellet-firing (ash removal): 12 ± 0.7% - wood-firing (maintenance): 11 ± 5.6% - recycled fuel-firing (maintenance): 4.8 ± 0.0% **Central nervous system disorders:** (*caused by combined effects of* *carbon monoxide*, *and hydrogen sulfide*) - recycled fuel-firing (ash removal): 17 ± 3.5% - wood-firing (ash removal): 6.8 ± 0.1%	/
Oesterhelweg & Püschel, 2008 [27]	Hydrogen sulfide gas exposure in a biogas plant	**Descriptive analysis:** - fatality: *n* = 4 - further health effects (among paramedics, nurses): mild intoxication symptoms (nausea, irritation of eyes, airways and skin)	/
Jumpponen et al., 2014 [30]	Multiple exposure to metals in biomass-fired power plants	**Effects of multiple exposure to metals:** (*only power plants and tasks reported that cause effects*, *percentages refer to Finnish Occupational Exposure Limits*; *mean* ± SD) **Cancer:** (*caused by combined effects of* *arsenic*, *beryllium*, *cadmium*, *and lead*) - recycled fuel-firing (ash removal): 2100 ± 1800% - peat-firing (ash removal): 230 ± 330% - wood-firing (ash removal): 56 ± 48% - peat-firing (maintenance): 21 ± 5% - recycled fuel-firing (maintenance): 15 ± 0.1% - wood-firing (maintenance): 50 ± 37% - pellet-firing (ash removal): 16 ± 5% **Central nervous system disorders:** (*caused by combined effects of* *manganese*, *lead*, *and selene*) - recycled fuel-firing (ash removal): 2000 ± 1800% - wood-firing (ash removal): 630 ± 630% - wood-firing (maintenance): 180 ± 180% - pellet-firing (ash removal): 110 ± 93% - peat-firing (ash removal): 73 ± 57% - peat-firing (maintenance): 69 ± 43% - recycled fuel-firing (maintenance): 14 ± 1% **Lower respiratory tract irritation:** (*caused by combined effects of* *beryllium*, *cadmium*, *manganese*, *and selene*) - wood-firing (ash removal): 660 ± 660% - wood-firing (maintenance): 180 ± 170% - recycled fuel-firing (ash removal): 150 ± 110% - pellet-firing (ash removal): 120 ± 94% - peat-firing (maintenance): 76 ± 42% - peat-firing (ash removal): 69 ± 45% - recycled fuel-firing (maintenance): 22 ± 2% **Upper respiratory tract irritation:** (*caused by combined effects of* *aluminium*, *arsenic*, *and selene*) - peat-firing (ash removal): 320 ± 360% - recycled fuel-firing (ash removal): 320 ± 280% - wood-firing (ash removal): 120 ± 110% - wood-firing (maintenance): 99 ± 110% - peat-firing (maintenance): 24 ± 8% - pellet-firing (ash removal): 6 ± 4% - recycled fuel-firing (maintenance): 5 ± 3%	/
Schlunssen et al., 2011 [26]	Working in a (straw, wood) power plant	**Association “Working in a …-power plant and … ”:** (*logistic regression; control group: working in a conventional power plant; rhinitis*, *daily coughing*, *asthma symptoms*, *current asthma*, *work-related asthma*/*wheeze; adjusted for smoking*, *atopy; work-related nose symptoms adjusted for smoking*, *atopy*, *age*) - **rhinitis**: straw: OR = 1.0 (95% CI: 0.4–2.4), wood: OR = 0.7 (95% CI: 0.3–1.5) - **daily coughing**: straw: OR = 0.8 (95% CI: 0.4–1.7), wood: OR = 1.6 (95% CI: 0.8–3.0) - **asthma symptoms**: straw: **OR = 7.6** (95% CI: 1.4–40.4), wood: OR = 2.2 (95% CI: 0.4–12.8) - **current asthma**: straw: OR = 0.8 (95% CI: 0.1–4.9), wood: OR = 0.4 (95% CI: 0.1–2.6) - **work-related asthma/wheeze**: straw: OR = 3.3 (95% CI: 0.6–17.9), wood: OR = 2.2 (95% CI: 0.4–11.9) - **work-related nose symptoms**: straw: OR = 2.3 (95% CI: 0.8–6.4), wood: OR = 1.5 (95% CI: 0.5–3.9) **Statistical significant associations:** (*statistical non-significant associations:* see Schlunssen et al., 2011 [26]) - “endotoxin, most exposed—work-related nose symptoms”: **OR = 3.1** (95% CI: 1.1–8.8) - “endotoxin, most exposed in a straw power plant—asthma symptoms”: **OR = 8.7** (95% CI: 1.1–71.4) - “dust, most exposed—work-related nose symptoms”: **OR = 3.2** (95% CI: 1.1–9.2) - “dust, most exposed—asthma symptoms”: **OR = 9.4** (95% CI: 1.7–52.0) - “fungi, most exposed in a straw power plant—asthma symptoms”: **OR = 17.8** (95% CI: 2.3–137) - “fungi, most exposed in a straw power plant—work-related asthma/wheeze”: **OR = 7.4** (95% CI: 1.1–48.1) - “Aspergillus fumigatus, moderately exposed in a wood power plant—work-related asthma/wheeze”: **OR = 4.0** (95% CI: 1.6–26.2) - “Aspergillus fumigatus, moderately exposed in a straw power plant—work-related nose symptoms”: **OR = 5.5** (95% CI: 1.2–25.2) - “Aspergillus fumigatus, most exposed in a straw power plant—work-related nose symptoms”: OR = 4.2 (95% CI: 1.0–18.3)	/
**Studies from Residential Settings**			
**Study**	**Exposure**	**Results**	**Influencing Factors**
Claeson et al., 2013 [24]	Odorous air pollution in the vicinity of a biofuel facility	**Association “odorous air pollution and … ”:** (*Spearman correlation coefficient*) - annoyance: **r = 0.36** (*p* < 0.05) - symptoms: **r = 0.08** (*p* < 0.05) (- perceived pollution: **r = 0.47** (*p* < 0.01)) (- health risk perception: **r = 0.33** (*p* < 0.01)) **Association “perceived pollution and …”:** - annoyance: **r = 0.69** (*p* < 0.01) - symptoms: **r = 0.06** (p > 0.05) - (health risk perception: **r = 0.49** (*p* < 0.01)) **Association “health risk perception and …”:** - annoyance: **r = 0.57** (*p* < 0.01) - symptoms: **r = 0.12** (*p* < 0.01)	**Mediators:** (*Spearman correlation coefficient*, *path analysis*) - perceived pollution - health risk perception (see Section 3 Results)
		**Association “annoyance and …”:** - symptoms: **r = 0.11** (*p* < 0.01) **Association …:** (*path analysis*) - “odorous air pollution and perceived pollution”: **r = 0.47** (*p* < 0.001) - “perceived pollution and annoyance”: **r = 0.55** (*p* < 0.001) - “perceived pollution and health risk perception”: **r = 0.49** (*p* < 0.001) - “health risk perception and annoyance”: **r = 0.30** (*p* < 0.001) - “health risk perception and symptoms”: **r = 0.12** (*p* < 0.01)	
Juntarawijit, 2013 [25]	Living near biomass power plants	**Association “Living near biomass power plants and …”:** (*no details on statistical methods*, *exposure group I: 0*–*0.5 km*, *II: 0.5*–*1.0 km*, *control group: > 1 km*) **Chronic diseases:** - **allergy**: I: **OR = 2.4** (95% CI: 1.5–4.0), II: OR = 0.8 (95% CI: 0.4–1.4) - **asthma**: I: OR = 1.2 (95% CI: 0.6–2.5), II: OR = 2.1 (95% CI: 1.0–1.44) - **heart disease**: I: OR = 1.1 (95% CI: 0.5–2.7), II: OR = 0.7 (95% CI: 0.2–2.0) - **COPD**: I: OR = 2:7 (95% CI: 1.0–8.4), II: OR = 0.4 (95% CI: 0.0–2.2) - **tuberculosis**: I: OR = 1.8 (95% CI: 0.4–7.5), II: OR = 1.0 (95% CI: 0.2–6.1) - **cancer**: I: OR = 0.3 (95% CI: 0.1–1.7), II: OR = 0.5 (95% CI: 0.1–2.5) **Health symptoms:** - **itching/rash**: I: **OR = 7.2** (95% CI: 4.2–12.5), II: OR = 1.1 (95% CI: 0.5–2.1) - **eye irritation**: I: **OR = 5.3** (95% CI: 3.0–9.1), II: OR = 1.7 (95% CI: 0.9–3.3) - **cough**: I: **OR = 3.9** (95% CI: 2.3–6.6), II: OR = 0.8 (95% CI: 0.4–1.6) - **stuffy nose**: I: **OR = 8.5** (95% CI: 4.4–16.4), II: OR = 2.1 (95% CI: 1.0–4.6) - **allergic symptoms**: I: **OR = 2.7** (95% CI: 1.6–4.5), II: OR = 0.3 (95% CI: 0.1–0.7) - **sore throat**: I: **OR = 2.5** (95% CI: 1.5–4.4), II: OR = 0.7 (95% CI: 0.3–1.4) - **difficulty breathing**: I: **OR = 6.7** (95% CI: 3.3–13.6), II: **OR = 3.1** (95% CI: 1.4–6.9)	/
**Studies from Occupational and Residential Settings**			
**Study**	**Exposure**	**Results**	**Influencing Factors**
Sovacool et al., 2015 [28]	Biomass energy production and distribution	**Fatalities worldwide** (*1874*–*2014*): - *n* = 97 **Normalized risk/TWh** (*1990*–*2013*): - 0.0164	/

CI: confidence interval, COPD chronic obstructive pulmonary disease, km: kilometer, *n:* sample size, **OR**: odds ratio, *p:p*-value, **r**: correlation coefficient, SD: standard deviation, **ThW**: terawatt hour. Bold, italics and underline are for better illustration.

**Table 4:** Methodological assessment of included studies.

Study	Reporting Quality	Selection				Information Bias		Confounders Considered?	Ethical Issues			Generali-Zation
		Sampling	Response	Eligibility of Comparison Group	Bias	Exposure	Outcome		Conflict of Interest	Funding	Ethics Committee	
**Studies from Occupational Settings**												
Basinas et al., 2012 [23]	+	+	+	+	+	+	+ (atopy) − (all other symptoms)	+	+	+	+	unclear
HSE, 2015 [31]	−	−	NA	−	−	−	+	−	unclear	unclear	unclear	−
Jumpponen et al., 2013 [29]	−	NA	NA	unclear	unclear	+	unclear	unclear	unclear	+	NA	+
Jumpponen et al., 2014 [30]	−	NA	NA	unclear	unclear	+	unclear	unclear	unclear	+	NA	+
Oesterhelweg & Püschel, 2008 [27]	+	NA	NA	−	+	−	+	−	unclear	unclear	NA	−
Schlunssen et al., 2011 [26]	+	+	+	+	+	+	+	+	+	+	+	unclear
**Studies from Residential Settings**												
Claeson et al., 2013 [24]	+	+	+	−	+	−	unclear	unclear	+	+	unclear	−
Juntarawijit, 2013 [25]	+	unclear	unclear	+	unclear	−	unclear	unclear	unclear	+	unclear	−
**Studies from Occupational and Residential Settings**												
Sovacool et al., 2015 [28]	+	NA	NA	+	unclear	+	+	−	unclear	unclear	NA	+

NA: not applicable, plus-symbol (+): high methodological quality, minus-symbol (−): low methodological quality.
